# Learning How to Run a Lab: Interviews with Principal Investigators

**DOI:** 10.1371/journal.pcbi.1003349

**Published:** 2013-11-07

**Authors:** Theodore Alexandrov, Philip E. Bourne

**Affiliations:** 1 Departments of Biochemistry and Mathematics, University of Bremen, Bremen, Germany; 2 SCiLS GmbH, Bremen, Germany; 3 Steinbeis Innovation Center SCiLS Research, Bremen, Germany; 4 Skaggs School of Pharmacy and Pharmaceutical Science, University of California San Diego, La Jolla, California, United States of America

A research lab is the workhorse of modern science. Running a research lab, whether it is a wet lab, dry lab, or both, is a complex and costly endeavor, which requires a talented team, solid funding, lab space, and a principal investigator (PI). The success of a lab is normally associated with its PI and, indeed, very much dependent on his or her scientific achievements (hence vision), productivity, and, importantly, management skills. Management skills are essential, because the individual contribution of the PI alone is insufficient to make a lab flourish. Ultimately, it is the ability of the PI to envision, manage, motivate, and lead the team that makes this workhorse healthy and productive.

*“Running a research lab is a complex and costly endeavor.”*

With all the complexity of this goal, and the size and turnover of a lab being comparable to that of a small company, it is surprising that scientific PIs do not normally have formal management training, and, instead, are typically self-made managers. They learn all the necessary management skills by inheriting them from their mentors during their graduate and postdoc career training and/or by their own trial and error. The lack of proper training provided is surprising, especially considering the time and expense necessary to succeed in a science career. This becomes even more astonishing knowing that, in other areas, professional teamwork and management is typically studied for decades and competent management education is offered. Given the parallels between running a lab and directing a small company, the former may be even more complex. Companies can use financial incentives. Scientific labs must rely mostly on career advancement and quality of the research experience.

*“Scientific PIs do not normally have formal management training, and, instead, are typically self-made managers.”*

Science could benefit from adopting management approaches, techniques, and expertise created in other fields of professional teamwork. The closest field to science is possibly software engineering and development. In its structure and spirit, a small software company very much resembles a scientific lab: It is a small motivated group of highly-educated professionals creating nonmaterial products with teamwork centered around collaboration between individuals. Science could benefit from using frameworks for project management, process control, issue tracking, and time management, which have been undergoing development in IT for decades, such as modern approaches of agile software development describing collaboration of self-organizing cross-functional teams.

For a young PI at the beginning of his or her independent career, it is especially important to learn more about running a successful lab and to escape common pitfalls [1]. Many questions pave the way: how to motivate team members; how to organize communication; how to handle conflicts inside the team; how to deal with increasing flows of information, the growing number of emails, university commitments, and publications. Unfortunately, no HowTo's are available where these questions are considered, with respect to lab management. Getting the advice of experienced PIs through discussions is great but, unfortunately, is almost the only way of getting answers to these questions. The amount of information published on this topic is miserable.

*“For a young PI at the beginning of his or her independent career, it is especially important to learn more about running a successful lab.”*

So, experienced PIs who have succeeded have much to offer, not just in describing what they do that is right in managing and growing a lab, but also by what they have done that is wrong. Further, times change—institutional structures change, technologies change, attitudes change. So as in all things, the old can learn from the young.

Our motivation then, in creating the collection of publications “About My Lab” for *PLOS Computational Biology*, is to provide a dialog from which we can all learn from each other. In doing so, we will raise awareness of the role of management in science, and provide an open platform for disseminating experience and opinions on this topic. *PLOS Computational Biology*, with its commitment to open knowledge, a large interdisciplinary audience, opportunities for readers to provide their comments and opinions, and modern ways of knowledge dissemination, is the perfect stage to raise these questions. Following the success of the personal format of the “Ten Simple Rules” collection, which has just celebrated 1,000,000 downloads, we will be sharing personal stories; stories that can be heard at a conference dinner and which later influence our decisions on key matters. We invite you to take part in this world-wide conference dinner, to share your opinion. and to listen to stories told by others. At the very least they will make for good gossip, and admit it or not, we all like good gossip.

*“We invite you to take part in this world-wide conference dinner.”*
